# Takayasu’s Arteritis in a 33-Year-Old Male

**DOI:** 10.7759/cureus.14557

**Published:** 2021-04-19

**Authors:** Raoon Khan, Anum Arif, Syed Hashim Ali Inam, Bismah Riaz, Hamza Jamil

**Affiliations:** 1 Internal Medicine, Combined Military Hospital Lahore Medical College and Institute of Dentistry, Lahore, PAK; 2 Vascular Surgery, Combined Military Hospital Lahore Medical College and Institute of Dentistry, Lahore, PAK; 3 Internal Medicine, Army Medical College, Rawalpindi, PAK

**Keywords:** vasculitis, takayasu disease, takayasu's arteritis, large vessel vasculitis

## Abstract

Takayasu’s arteritis (TA), commonly referred to as “pulseless” disease, is a large-vessel inflammatory vasculitis most commonly affecting the aorta and its major branches. Due to its irregular nature, it has the propensity to involve any organ system thus leading to a wide spectrum of clinical features. Most patients affected by TA are females in their second or third decades of lives. Our case is of a 33-year-old male who presented with sudden onset of hypertension for which he was prescribed antihypertensives. Over the next few weeks, he had multiple visits to the emergency department for a variety of different symptoms including fever, myalgias, left arm numbness, and persistence of hypertension. His CT aortogram showed multi-vessel narrowing including that of the celiac axis, superior mesenteric, renal, and internal iliac arteries with right atrophic kidney. At this time, a diagnosis of TA was made and he was started on oral corticosteroid and immunosuppressant therapy and continued to be treated as an outpatient. Nearly five years after his initial symptoms, he presented to the Emergency for acute abdomen, severe vomiting, and constipation, at which time an emergency laparotomy was done and peritonitis was found. A CT angiogram of the abdomen done after this procedure showed tight stenosis of the inferior mesenteric artery (IMA) and proximal stenosis of the left renal artery. He is currently planned for left renal artery and IMA stenting. Our case highlights the important characteristics of TA in male patients and how they differ from females. It also focuses on the importance of early initial workup and diagnosis and the need for a multi-disciplinary team when handling any patient with TA.

## Introduction

The first case of Takayasu’s arteritis was presented in 1908 by Dr. Mikito Takayasu, a Japanese professor of Ophthalmology who presented the findings of a 22-year-old woman with "peculiar changes in the central retinal vessels," exhibiting symptoms of blurry vision and occasional redness of conjunctiva since 1905 [1]. With contributions of similar case reports by many other great minds, the disease today is known as a "pulseless" disease and is characterized as a rare granulomatous vasculitis of medium and large-sized vessels, with a strong predilection for the aorta and its major branches including the coronary, carotid, pulmonary and renal arteries [2]. Originating in Japan, the disease is now recognized worldwide with the majority of cases in Asia, Africa, and Latin America and an incidence estimated to be 2.69/million/year in Asia [3]. It is most notorious for affecting young females during the second or third decades of life, however, it has also been reported in the pediatric population as common vasculitis [4]. The precise etiology remains unknown but various studies have attributed infections and autoimmunity as plausible causes. Moreover, a genetic link has also been identified [2]. Ultimately, the involved artery undergoes inflammation, which leads to subsequent fibrosis, thickening of the intima, stenosis, occlusion, and thrombus formation [3]. This, in turn, may lead to various pathological effects including end-organ ischemia and/or aneurysm/dilatation of the involved vessel. The disease has the propensity to involve any major organ system resulting in a plethora of clinical manifestations [5].

## Case presentation

A 33-year-old male with no known comorbidities presented to the medical clinic with a sudden onset of hypertensive crisis for a few hours’ duration. He was given antihypertensive medication including nifedipine (60 mg, once daily) and candesartan (16 mg, once daily) which allowed for temporary relief of symptoms.

**Figure 1:**
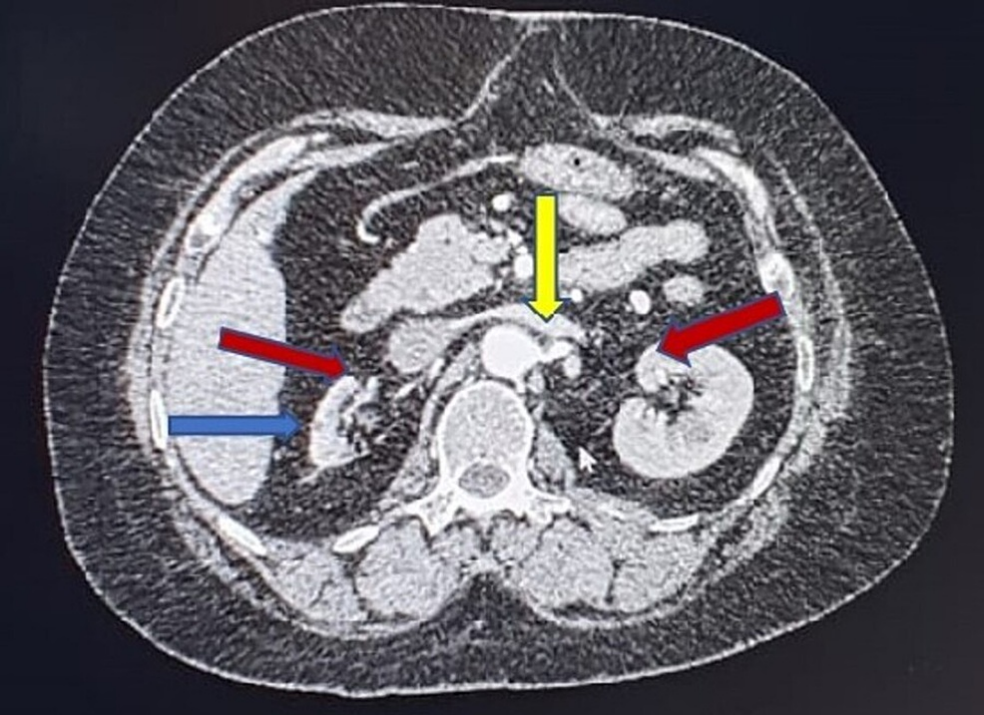
CT angiogram abdomen (axial view). Blue arrow indicates atrophic right kidney. Red arrows indicates bilateral renal artery stenosis. Yellow arrow indicates stenosis of inferior mesenteric artery origin.

**Figure 2:**
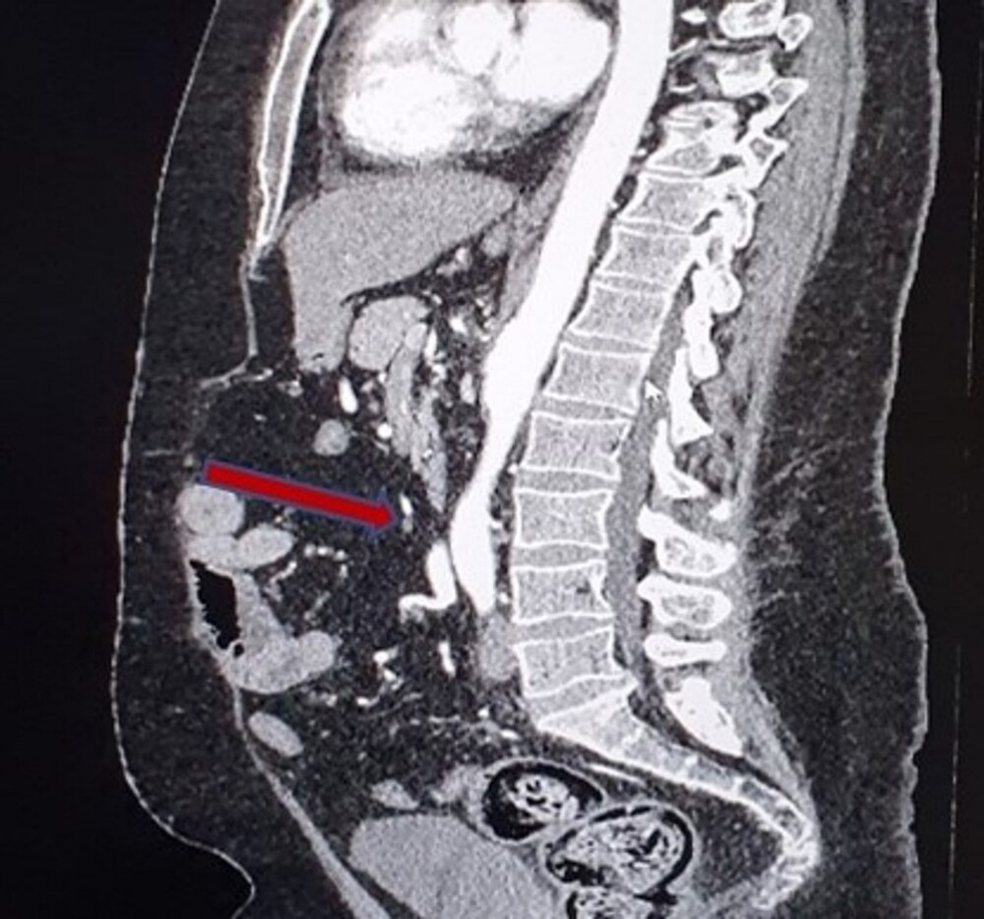
CT angiogram (sagittal view). Red arrow indicates narrowing at the origin of the common iliac artery.

Over the next couple of months, he presented with similar complaints on multiple occasions. He was referred to a nephrologist and a CT angiogram of the abdomen was done which disclosed diffuse thickening of the abdominal aorta from D11-L2 and mild dilatation from D11-L2. Segmental areas of thickened wall and mild to severe narrowing were seen in parts of the celiac axis, superior mesenteric, renal, and internal iliac arteries. The right kidney's size was shrunken due to renal artery stenosis. This is illustrated in Figure 1 and Figure 2.

At this time, he also started having mild pain and numbness in his right arm and leg. The patient was admitted and a CT angiography arm was done which revealed tight stenosis of the right subclavian artery. A preliminary diagnosis of Takayasu’s arteritis was made at this time and the patient was started on deltacortil (60 mg/day), and azathioprine (200 mg/day) tablets. The patient took these medications in addition to his anti-hypertensives and symptoms remained controlled. No referral to a vascular surgeon was made at that time.

Throughout the next two years, he started having intermittent bouts of high-grade fever associated with chills, rigors, and severe sweating. This was accompanied by severe arthralgias and myalgias. He presented to local medical clinics where he received symptomatic management.

In 2019, his renal scintigraphy was done which showed a non-functioning right kidney and normal functioning left kidney. Later that year, he landed in the emergency department with high-grade fever accompanied by severe generalized abdominal pain, vomiting, and constipation. An emergency laparotomy was planned for his peritonitis. Intra-operative findings included segmental areas of gangrenous bowel 18 inches from the duodenojejunal (DJ) flexure and in most of the ileum. An ileostomy was made. Two days later, he had a re-look laparotomy at which time all the remaining ileum was also gangrenous and so was excised and a jejunostomy and a mucous fistula were made.

In late 2020, the patient came for a routine check-up. A repeat CT angiogram (abdominal aorta and pelvis) was done which revealed normal opacification of the thoracic and abdominal aorta but disease progression in the celiac trunk and proximal superior mesenteric artery, which both showed complete occlusion. There was also tight ostial stenosis of the inferior mesenteric artery followed by diffuse post stenotic dilatation. Furthermore, there was severe proximal segment stenosis of the right renal artery with slender branches, and the size of the right kidney was significantly small. Severe proximal stenosis of the left renal artery were also seen with compensatory hypertrophy of the left kidney. Lastly, there was complete occlusion of the right internal iliac artery with subtotal ostial stenosis of the left internal iliac artery. 

A few months later, he experienced repeated episodes of postprandial epigastric pain and was diagnosed with acute cholecystitis due to short bowel syndrome, which was managed conservatively. Currently, the patient continues to have elevated blood pressure despite the routine use of anti-hypertensives and steroids. He has been visited by the vascular surgery team who have planned for stenting of left renal and inferior mesenteric arteries, followed by stoma reversal.

## Discussion

Takayasu’s arteritis is a rare vasculitis, affecting 1-2 million worldwide [6]. The typical patient was once identified as a young female under 40 years, of East Asian descent [2]. Over time, it was observed that TA is prevalent in both genders, at any age, belonging to any ethnic group [2]. The youngest patient described was six months old and the oldest was 75 years old [6, 7]. Most patients however are in their third decade of life and 90% of these patients are females [4]. Our case presents the rare findings of a young Pakistani male whose initial symptoms appeared at 33 years of age. According to the American College of Rheumatology, our patient met three of the six criteria for the diagnosis of the disease [2]. These include age <40 years, claudication of the extremities, and narrowing of the aorta or its primary branches on the arteriogram.

Although the causes of TA remain largely unknown, three etiological factors have been implicated as follows: 1) cell-mediated autoimmunity involving γδT-cells, αβT-cells (CD4 and CD8), natural killer cells, and anti-endothelial antibodies; 2) infections including *Mycobacterium tuberculosis* and certain viruses; 3) genetics, as the disease has been known to manifest monozygotic twins [2]. Furthermore, different ethnic groups have distinctive human leukocyte antigen (HLA) types, giving rise to various phenotypes [5]. In all cases, the result is a transmural fibrous thickening of the arterial walls, which may further result in occlusion, stenosis, or thrombus formation, all of which inevitably cause end-organ ischemia [2]. In some cases, injury to elastic fibers may lead to aneurysm formation [6]. The most common pattern of the disease involves the entire aorta and primary branches both above and below the diaphragm [2]. Of these, the subclavian artery (Left>Right) is most commonly affected, followed by common carotid (Left>Right), renal, vertebral, and innominate arteries [2]. Our patient also had tight stenosis of the right subclavian artery along with narrowing in parts of the celiac axis, superior mesenteric, renal, and internal iliac arteries.

In 1994, the Takayasu Conference in Tokyo proposed an angiographic classification that differentiates patients into six subgroups according to angiographic involvement. According to this classification, our patient was Type V [8]. This can be linked to a study comparing the distribution of TA lesions in both males and females of Indian ethnicity which found that females have a higher tendency for involvement of aortic arch and its branches, whereas, in males, abdominal aorta involvement is much higher [8]. However, there have also been case reports of male patients such as a 22-year-old Pakistani male and a 57-year-old Bangladeshi man, both having lesions of branches from the aortic arch only (Type I) [7, 9]. Variations in vessel involvement also exist between countries. A study comparing Japanese and Indian patients showed that Japanese patients were mostly (96%) female and had involvement of aortic arch and its main branches. Contrastingly, Indian patients included a higher prevalence of males (37%), and these patients had an increased involvement of abdominal aorta and renal vessels [10]. This reflected the findings in our patient.

The greatest obstacle associated with a diagnosis of TA is the vast spectrum of symptomatology that a patient may present with [11]. The disease may be entirely asymptomatic, or it may present with a plethora of symptoms, each reflecting the area of vessels involved [5].

For simplification, two phases of the disease have been identified as follows: 1) Acute “pre-pulseless” phase characterized by non-specific inflammatory features such as fever, malaise, myalgias, arthralgia, and weight loss; and 2) chronic phase, caused by the progression of inflammation and formation of vascular lesions and characterized by symptoms according to the vessels involved [4]. Most affected patients experience overlapping of the two phases and their corresponding symptoms [2]. Our patient initially presented with sudden onset of hypertension due to renal artery stenosis. This is the presenting symptom in 33-83% of TA patients [5]. Renal artery involvement is especially common in Indian patients, who usually present with hypertension as their initial complaint [10]. His remaining symptoms appeared gradually over the next few months, which included right arm numbness and fever with myalgias and arthralgias. A few years later, he experienced post-prandial epigastric pain. In a study comparing clinical features between males and females, it was observed that no significant difference exists except males exhibit abdominal pain more frequently than females, which was observed in our patient [12]. Another study comparing the two genders found that males present with symptoms of hypertension, bruits, and retinopathy much more as compared to females [8]. In a case report with the 22-year-old male, the patient’s cardiovascular examination showed muffled heart sounds and a carotid bruit along with constitutional symptoms [9]. The 57-year-old male presented with a decline in visual acuity for three months which was associated with generalized fatigue as well as pain and numbness in the extremities [7].

When symptoms lead to suspicion, the gold standard for diagnosis is angiography [5]. CT angiography (CTA) is more commonly employed as it allows the study of wall inflammation [2]. NMR (nuclear magnetic resonance) angiography and 18F fluorodeoxyglucose positron emission tomography (18F FDG-PET) are recent, more favorable techniques as they aid in the diagnosis of TA even in the pre-stenotic phase by detecting structural wall changes [3, 13]. They are also useful for evaluating the response to treatment in TA [2]. Laboratory tests including acute phase reactant markers (C-reactive protein) or erythrocyte sedimentation rate (ESR) may be done but are non-specific for TA [3]. Doppler ultrasonography may reveal stenosis of affected arteries but it is also nonspecific in TA [5]. Chest radiography, coronary angiography, and lung perfusion tests may be warranted to rule out associated, less common symptoms [2]. Diagnosis of TA is a multifaceted process that involves anatomic involvement and clinical symptoms and prognosis. Most patients have a median delay of 15 months between onset of symptoms and diagnosis of disease [2]. Our patient had approximately a six-month delay. It has also been reported that 91% of patients claim to have been seen by at least one doctor before diagnosis [13]. Furthermore, most rural hospitals do not have the availability of non-invasive imaging modalities such as magnetic resonance angiography (MRA), CTA, and FDG-PET [13].

Medical therapy includes steroids which is the mainstay of treatment and to which approximately half of the patients respond [5]. Other patients may require immunosuppressants including cyclophosphamide, azathioprine, or methotrexate [2]. Along with TA itself, it is important to treat associated symptoms simultaneously. In a case report with the 57-year old male, he required pan-retinal photocoagulation of both eyes [8]. In some cases, the symptoms may persist and surgical intervention, including endovascular revascularization procedures, are necessary [8]. Indications for surgery include hypertension due to renal artery stenosis, severe coronary artery or cerebrovascular disease, stenotic lesions resulting in critical limb ischemia, and aneurysms at risk of rupture [14]. In such cases, consultation with the vascular surgery team is required. This was done for our patient who is planned for stenting of left renal and inferior mesenteric arteries. Although many studies have shown the benefit of surgery to correct stenosis, these procedures have a high failure rate, especially when undertaken during a period of inflammatory activity [7].

## Conclusions

TA is a disease with a wide spectrum of non-specific early and late manifestations that are regularly being overlooked by healthcare professionals as diseases such as systemic lupus erythematosus, rheumatoid arthritis, sarcoidosis, Buerger's disease, and various other systemic diseases. Additionally, due to lack of its awareness, many patients don’t seek medical treatment until signs of the "pulseless" phase occur, at which point significant morbidity has already occurred. This can be attributed to the rarity of this disease and the fact that only a handful of work has been done on it. Further, when a patient is diagnosed, it is important to counsel the patient regarding routine check-ups since this disease has the potential to worsen and result in more deleterious effects. In our patient, the CT angiogram abdomen which was done five years after initial diagnosis showed stenosis of inferior mesenteric and left renal arteries as well as occlusion of bilateral internal iliac arteries - findings that were not present in his earlier tests. As a result, he started experiencing severe post prandial epigastric pain which ultimately led to peritonitis and the need for invasive surgical intervention. Lastly, patients of TA should be handled by a multidisciplinary team including general practitioners, rheumatologists, cardiologists, ophthalmologists, nephrologists and vascular surgeons. Due to the disease’s unpredictable nature, each specialty should be aware of the patients affected and should warrant any investigations necessary to rule out manifestations respective of their field.
